# Retention of locally injected human iPS cell-derived cardiomyocytes into the myocardium using hydrolyzed gelatin

**DOI:** 10.1038/s41598-025-87885-w

**Published:** 2025-02-07

**Authors:** Jun Iida, Kazuki Kotani, Kozue Murata, Keisuke Hakamada, Wusiman Maihemuti, Yoshinobu Mandai, Yosuke Hiraoka, Kenji Minatoya, Hidetoshi Masumoto

**Affiliations:** 1 Clinical Translational Research Program, RIKEN Center for Biosystems Dynamics Research, 2-2-3 Minatojimaminami-cho, Chuo-ku, Kobe, 650-0047 Japan; 2 Department of Cardiovascular Surgery, Graduate School of Medicine, Kyoto University, Kyoto, Japan; 3 Biomedical Department, R&D Center, Nitta Gelatin Inc, Yao, Japan

**Keywords:** Cardiovascular biology, Biomaterials

## Abstract

This study explored the impact of hydrolyzed gelatin (HG) concentration on the retention and therapeutic efficacy of human iPS cell-derived cardiomyocytes (hiPSC-CMs) when injected into the myocardium. The solubility of HG allows precise control over its concentration, influencing the distribution and leakage of injected solutions, which may affect therapeutic outcomes. Using both ex vivo and in vivo rat models, we investigated how varying HG concentrations affect the retention of solution and diffusion within the myocardium. In ex vivo static rat hearts, 10% HG minimized leakage but allowed significant diffusion. However, in pulsating in vivo hearts, 20% HG provided the best retention. In a rat myocardial infarction model, hiPSC-CMs suspended in 20% HG resulted in the highest cell retention. Echocardiogram showed a significant increase in the ejection fraction two weeks after transplantation compared to before transplantation. Additionally, cardiac magnetic resonance imaging (MRI) revealed that the ejection fraction was significantly higher than that of the sham group four weeks after transplantation. These findings suggest that optimizing HG concentration is crucial for enhancing the retention and therapeutic efficacy of hiPSC-CM transplants in treating heart disease.

**Supplementary Information:**

The online version contains supplementary material available at 10.1038/s41598-025-87885-w.

## Introduction

The number of heart failure patients continues to increase worldwide^1^. On the other hand, heart transplantation, which is considered effective for the most severe heart failure patients, faces the issue of donor shortages^2^, and ventricular assist devices (VAD) also have problems, such as the risk of complications with long-term use^3^. In this context, cardiac regenerative therapy is expected to be one of the methods to solve these problems^4–7^. Among cardiac regenerative therapies, in the method where cardiomyocytes (CMs) created by inducing differentiation using pluripotent stem cells such as embryonic stem cells or induced pluripotent stem (iPS) cells are transplanted into the myocardial tissue of the diseased heart, it is important to increase the retention of the transplanted CMs within the tissue^8–10^. As one means to achieve this, optimizing additives/solvents has been examined^11,12^.

Gelatin and gelatin hydrogel have traditionally been used as an additive in cell injection therapy^13,14^. However, it undergoes sol-gel transition at 15 °C to 20 °C, causing changes in its physical properties, which makes fine-tuning its viscosity difficult^15,16^. The change in the physical properties of additives due to temperature may affect the effectiveness of transplantation. On the other hand, Hydrolyzed Gelatin (HG), which is made by hydrolyzing gelatin, is a low molecular weight polypeptide. Compared to traditional gelatin and gelatin hydrogel, HG is less prone to gelation^17^. This makes it less likely to undergo rapid changes in viscosity, allowing for finer adjustments in concentration and viscosity^18^. Due to these characteristics, along with its low antigenicity, HG is increasingly being recognized as a useful material in cell transplantation therapy^19^.

In our recent report, we demonstrated that altering the concentration of HG can reduce the leakage of the solution from the injection site^20^. This indicates that the amount of solution that can remain in the tissue when HG solution is added to the therapeutic agent and injected into the tissue is influenced by the concentration of HG. Based on this report, we investigated the effect of varying the concentration of HG added as an additive on the efficacy of intramyocardial injection of human iPSC-derived cardiomyocytes (hiPSC-CMs).

## Results

### Post-injection leakage of HG in ex vivo rat myocardium

**Fig. 1:**
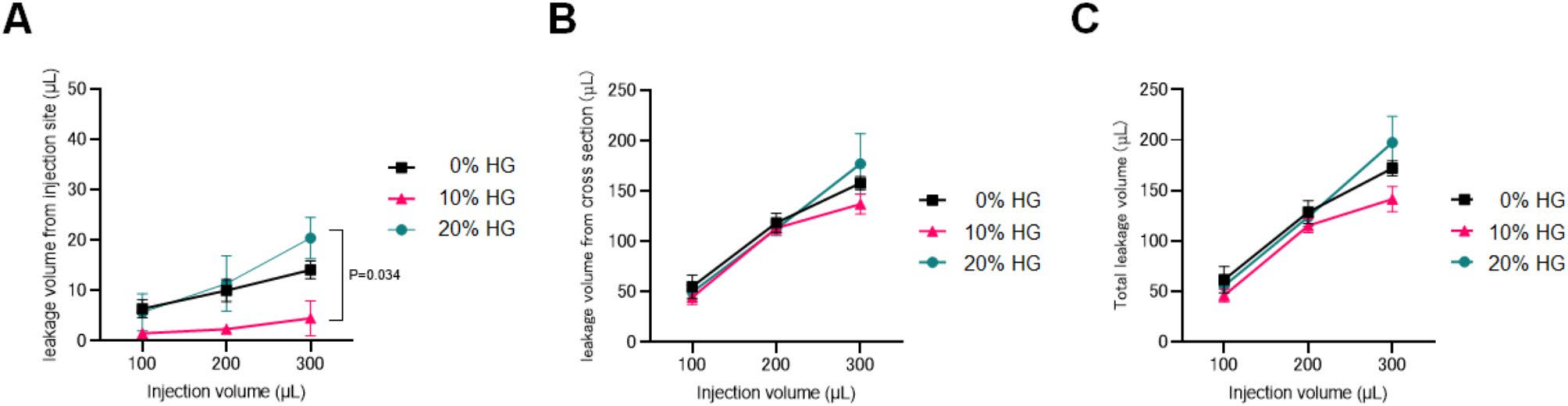
Ex vivo evaluation of the leakage of HG solution after injected into non-pulsating rat myocardium. (**A**) Change in leakage volume from the injection site for each HG concentration (*n* = 3 for each condition, mean ± SD, Kruskal-Wallis test with Dunn’s multiple comparison test). (**B**) Change in leakage volume from tissue cross-sections for each HG concentration (*n* = 3 for each condition, mean ± SD, Kruskal–Wallis test with Dunn’s multiple comparison test). (**C**) Total leakage volume from both the injection site and tissue cross-sections (*n* = 3 for each condition, mean ± SD, Kruskal–Wallis test with Dunn’s multiple comparison test).

First, we evaluated the dynamics of HG injection in static non-beating rat myocardial tissue ex vivo. The rat hearts were harvested and divided into two parts longitudinally after confirmation of spontaneous pulsation, then subjected to the injection of HG solution. The amount of leakage from the injection site with a 300 µL injection volume was smallest with 10% HG solution (10% HG vs. 20% HG, 4.5 ± 3.5 mg vs. 20.4 ± 4.1 mg, *P* = 0.034), showing an increase in leakage from the injection site in the order of 10% HG < 0% HG < 20% HG (Fig. 1A). This indicates that, consistent with previous studies^20^, changes in HG concentration affect the amount of leakage from the injection site in static rat myocardium. On the other hand, there was no significant difference in the amount of leakage from the tissue cross-section among different HG concentrations (0% HG vs. 10% HG vs. 20% HG, 158.0 ± 6.6 mg vs. 137.1 ± 9.8 mg vs. 177.2 ± 30.0 mg) (Fig. 1B). This suggests that the intramyocardial diffusion of HG solution within the non-beating myocardial tissue is not significantly influenced by the concentration of HG. It was also found that in fresh non-beating myocardial tissue, the leakage rate from the tissue cross-section was higher than that from the injection site. We evaluated the total leakage by combining the amounts from the injection site and the tissue cross-section. Although not statistically significant, there was a trend of increasing leakage with 10% HG < 0% HG < 20% HG for the 300 µL injection volume (Fig. 1C). These results indicate that in myocardial tissue injections of HG solution, the impact on the amount of solution retained within the tissue is more influenced by intramyocardial diffusion than the leakage from the injection site. This also suggests the need to consider intramyocardial diffusion within the tissue to assess local retention.

### Intramyocardial diffusion of injected HG within the beating rat myocardium

**Fig. 2:**
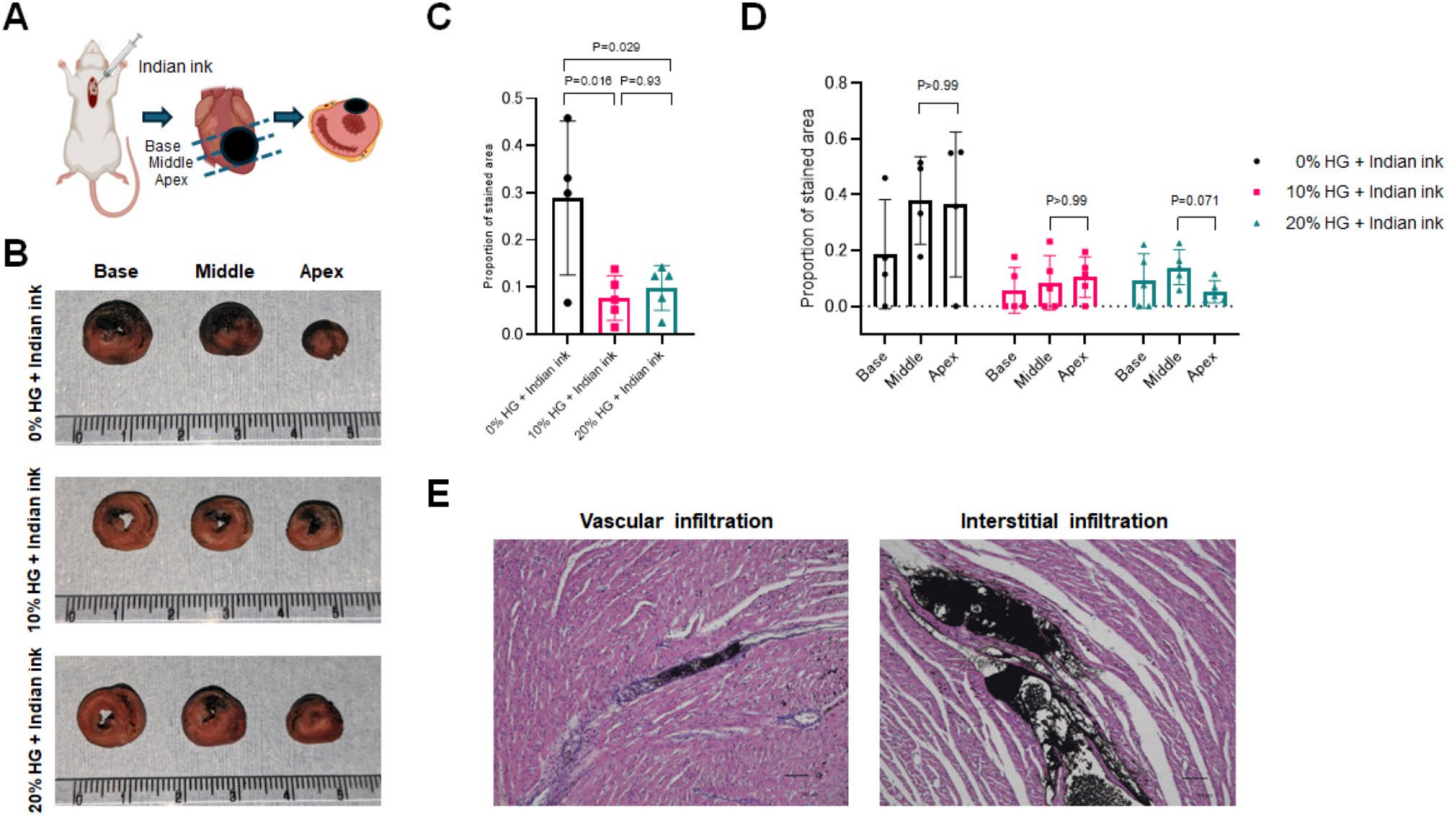
In vivo evaluation of tissue diffusion of injected Indian ink in pulsating myocardium. (**A**) Schematic diagram of the experiment. Indian ink was injected into the pulsating rat myocardium, and the stained area/ratio in three cross-sections (base, middle = injection level, apex) was compared and evaluated among three groups: Indian ink only (*n* = 4), 10% HG + Indian ink (*n* = 5), 20% HG + Indian ink (*n* = 5). (**B**) Representative macroscopic findings of ink-stained tissue sections of normal rat myocardium. Indian ink only (top), 10% HG + Indian ink (middle), 20% HG + Indian ink (bottom). The unit of the ruler is centimeter. (**C**) Ratio of stained area to total cross-sectional area. Kruskal–Wallis test with Dunn’s multiple comparison test. (**D**) Ratio of stained area to total cross-sectional area at each cross-sectional level. Kruskal–Wallis test with Dunn’s multiple comparison test. (**E**) Representative HE staining of 20% HG + India ink injected rat myocardium (in vivo; pulsating heart) one day after injection. Vascular infiltration (left) and interstitial infiltration (right). Scale bars = 100 μm.

Next, to evaluate intramyocardial diffusion of the injected solution in beating myocardium, we injected Indian ink solution into the beating rat myocardium in vivo and assessed the intramyocardial distribution of injected solution (Fig. 2A). We used normal heart tissue (non-infarcted heart) in this experiment to investigate the diffusion of the solution within the myocardium. The viscosity measurements showed that sufficiently diluted Indian ink shows no difference in viscosity and that adding such diluted Indian ink to the HG solution does not result in a viscosity difference compared to when it is not added (Table 1). For better visualization, we used 10% Indian ink solution in our experiments.

In the 0% HG + Indian ink group, extensive staining with the ink was optically observed in all planes, including the base, middle, and apex plane. In contrast, the stained area was narrower in the 10% HG + Indian ink group and the 20% HG + Indian ink group (Fig. 2B). The quantification of ink-stained areas on each cross-section was as follows: 0% HG + Indian ink vs. 10% HG + Indian ink vs. 20% HG + Indian ink, 55.9 ± 28.1 mm^2^ vs. 17.6 ± 12.1 mm^2^ vs. 21.6 ± 10.7 mm^2^, respectively. When normalized to left ventricular myocardial area, the area ratios were: 0% HG + Indian ink vs. 10% HG + Indian ink vs. 20% HG + Indian ink, 0.289 ± 0.163 vs. 0.077 ± 0.047 vs. 0.098 ± 0.047, respectively, indicating significantly wider distribution in the 0% HG + Indian ink group (vs. 10% HG + Indian ink, *P* = 0.016; vs. 20% HG + Indian ink, *P* = 0.029) (Fig. 2C). Quantification of the stained area ratio in sections at base, middle, and apex showed that 20% HG + Indian ink tended to have a larger stained area around the injection region (middle plane) compared to 10% HG + Indian ink (Fig. 2D). This suggests that 20% HG + Indian ink may more likely retain around the injected region in beating myocardium.

We evaluated how the injected Indian ink solution diffused within the beating myocardial tissue and found that the ink was partially distributed into the blood vessels in addition to the distribution within the interstitium between muscle fibers (Fig. 2E).

**Table 1:** List of viscosities of Indian ink solutions.

Solutions	Capillary flow time (s)	Calculated viscosity (mPa·s)
PBS (Phosphate-buffered saline)	99.62 ± 0.20	1.39
NS (Normal saline)	99.63 ± 0.16	1.39
DW (Distilled water)	98.02 ± 0.54	1.37
1% Indian Ink	100.53 ± 0.26	1.40
10% Indian Ink	114.28 ± 0.74	1.57
Indian Ink (original)	425.42 ± 21.39	5.30
10% HG	192.22 ± 0.45	2.50
20% HG	408.98 ± 0.73	5.10
10% HG + 10% Indian Ink	215.95 ± 0.29	2.78
20% HG + 10% Indian Ink	477.45 ± 12.54	5.92

### Histological evaluation for the retention of the transplanted hiPSC-derived CMs with HG in a rat MI model

**Fig. 3:**
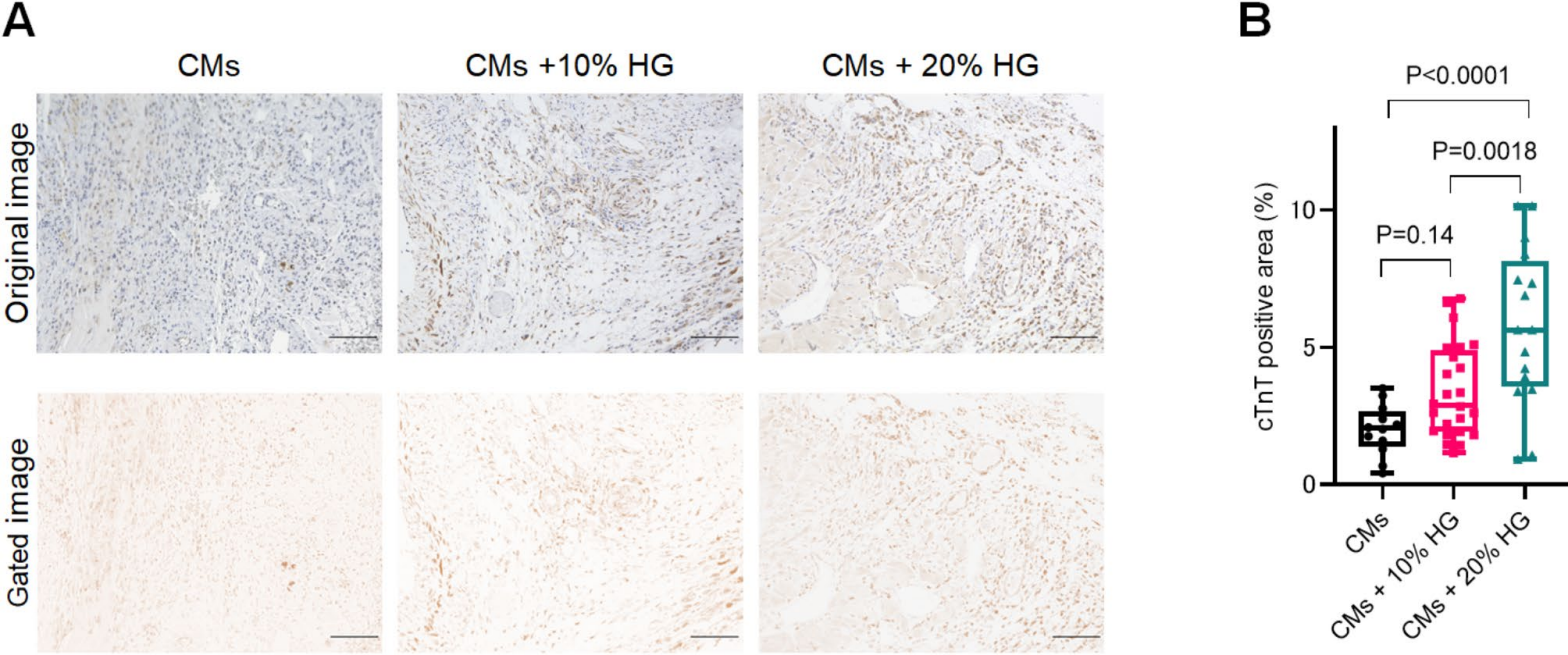
Evaluation of hiPSC-CM engraftment one week after injection in a rat MI model. (**A**) Representative immunostaining for cTnT. Scale bars = 100 μm. (**B**) Quantitative assessment of the cTnT-positive area percentage. CMs, *n* = 12; CMs + 10% HG, *n* = 20; CMs + 20% HG, *n* = 16 (All data points represent the number of analyzed fields of view, not the number of rats). One-way ANOVA with Tukey’s multiple comparison test.

Next, we examined how co-injection with various concentrations of HG affects the retention efficiency of injected hiPSC-CMs in the beating heart in vivo by performing a histological evaluation at the middle plane where the hiPSC-CMs were injected. In this experiment, there were cases where CM engraftment could not be confirmed one week after the injection; Consequently, the number of rats for which the cTnT-positive area ratio could be measured was as follows: CMs, *n* = 3; CMs + 10% HG, *n* = 5; and CMs + 20% HG, *n* = 4. The retained CMs were quantified as follows: CMs vs. CMs + 10% HG vs. CMs + 20% HG, 2.00 ± 0.93% vs. 3.39 ± 1.69% vs. 5.77 ± 2.90% (Fig. 3). There was a significantly higher retention in the CMs + 20% HG group (CMs vs. CMs + 20% HG, *P* < 0.0001, CMs + 10% HG vs. CMs + 20% HG, *P* = 0.0018). There was no significant difference of CM retention between CMs group and CMs + 10% HG group (*P* = 0.14).

### Transplantation of hiPSC-CMs with HG and functional evaluations in a rat MI model

**Fig. 4:**
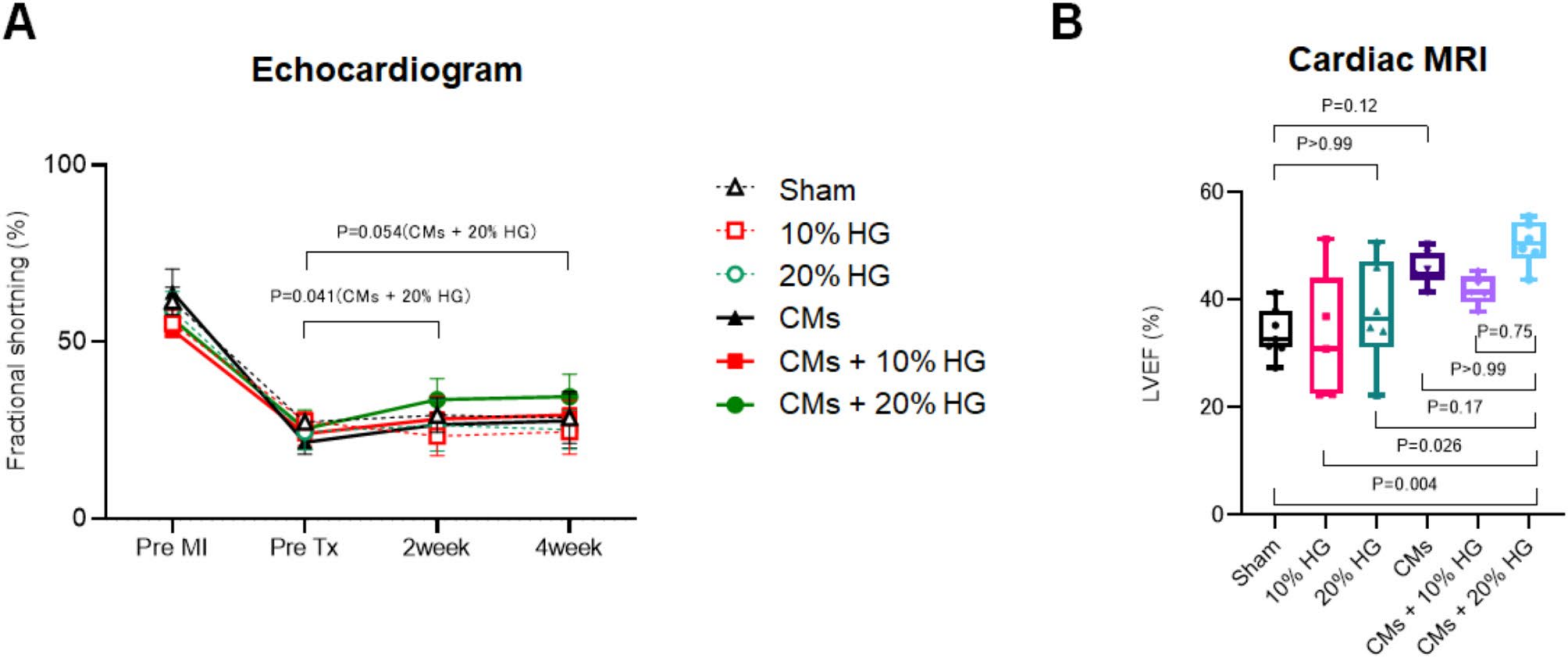
Cardiac function evaluation four weeks after hiPSC-CM injection in a rat MI model. (**A**) Temporal changes in fractional shortening up to four weeks post-transplantation as measured by echocardiogram. Sham, *n* = 7; 10% HG, *n* = 5; 20% HG, *n* = 6; CMs, *n* = 7; CMs + 10% HG, *n* = 5; CMs + 20% HG, *n* = 6. Mean ± SD, 2-way ANOVA with Tukey’s post hoc pairwise comparison. (**B**) Evaluation of the left ventricular ejection fraction (LVEF) by cardiac MRI four weeks post-transplantation. Sham, *n* = 7; 10% HG, *n* = 5; 20% HG, *n* = 6; CMs, *n* = 7; CMs + 10% HG, *n* = 5; CMs + 20% HG, *n* = 6. Mean ± SD, Kruskal–Wallis test with post-hoc group comparisons conducted using Dunn’s multiple comparison test.

We assessed cardiac function four weeks after intramyocardial injection of hiPSC-CMs at various concentrations of HG into a rat MI model. Systolic function assessed by FS (%) via echocardiogram was significantly improved only in the CMs + 20% HG group from PreTx to 2week (PreTx vs. 2 weeks, 25.5 ± 5.4% vs. 33.7 ± 6.0%; *P* = 0.041) (Fig. 4A). All parameters including LVDd, LVDs, and akinetic length are shown in Table 2.

At 4 weeks post-transplantation, cardiac MRI showed that CMs + 20% HG exhibited significantly higher LVEF compared to the sham group (sham vs. CMs + 20% HG: 33.8 ± 4.7% vs. 50.5 ± 4.1%; *P* = 0.004). However, there was no significant difference of LVEF between CMs group and sham control (sham vs. CMs: 33.8 ± 4.7% vs. 45.5 ± 3.0%; *P* = 0.12), or 20% HG and sham control (sham vs. 20% HG: 33.8 ± 4.7% vs. 37.6 ± 10.0%; *P* > 0.99) (Fig. 4B). All parameters including LVEDV and LVESV are shown in Table 3.

**Table 2:** Echocardiogram data after treatment.

Parameters (mean ± SD)	Group	Pre MI	Pre TX	2 W	4 W
LVEF (%)	Sham	93.3 ± 2.0	59.5 ± 3.5	61.8 ± 7.4	60.3 ± 11.1
	10% HG	89.7 ± 2.7	59.6 ± 4.0	52.0 ± 9.5	54.0 ± 10.1
	20% HG	91.8 ± 2.6	54.2 ± 8.8	56.8 ± 10.8	55.1 ± 8.9
	CMs	94.2 ± 3.1	49.3 ± 6.1	57.8 ± 5.0	58.5 ± 11.8
	CMs + 10% HG	88.6 ± 1.5	53.4 ± 5.8	59.6 ± 10.0	61.9 ± 5.9
	CMs + 20% HG	90.6 ± 1.9	55.7 ± 9.3	68.0 ± 7.9	68.8 ± 8.6
FS (%)	Sham	61.6 ± 3.9	27.4 ± 2.0	29.3 ± 4.9	28.6 ± 7.3
	10% HG	55.2 ± 3.8	27.6 ± 2.5	23.3 ± 5.6	24.6 ± 6.3
	20% HG	59.1 ± 5.1	24.6 ± 5.1	26.5 ± 7.3	25.2 ± 5.4
	CMs	64.1 ± 6.6	21.6 ± 3.4	26.6 ± 3.1	27.7 ± 7.7
	CMs + 10% HG	53.4 ± 2.1	24.1 ± 3.4	28.2 ± 6.4	29.4 ± 4.1
	CMs + 20% HG	56.6 ± 3.4	25.5 ± 5.4	33.7 ± 6.0	34.5 ± 6.3
LVDd (mm)	Sham	6.0 ± 0.4	6.5 ± 1.1	6.8 ± 0.9	6.9 ± 0.8
	10% HG	6.4 ± 0.7	6.6 ± 0.7	6.8 ± 1.5	7.2 ± 1.6
	20% HG	6.9 ± 0.6	6.8 ± 1.1	7.2 ± 1.1	7.1 ± 1.0
	CMs	6.4 ± 0.7	6.4 ± 1.2	6.8 ± 1.5	7.5 ± 1.1
	CMs + 10% HG	6.2 ± 0.6	7.2 ± 0.7	8.1 ± 0.9	7.6 ± 1.2
	CMs + 20% HG	6.4 ± 0.6	6.9 ± 0.6	7.1 ± 0.5	7.6 ± 0.8
LVDs (mm)	Sham	2.3 ± 0.3	4.7 ± 0.7	4.8 ± 0.7	5.0 ± 1.1
	10% HG	2.8 ± 0.2	4.7 ± 0.5	5.2 ± 0.9	5.4 ± 1.1
	20% HG	2.8 ± 0.3	5.1 ± 0.8	5.3 ± 1.0	5.3 ± 1.0
	CMs	2.3 ± 0.6	5.0 ± 0.9	5.0 ± 1.0	5.4 ± 0.6
	CMs + 10% HG	2.9 ± 0.4	5.5 ± 0.5	5.8 ± 0.9	5.3 ± 0.6
	CMs + 20% HG	2.8 ± 0.3	5.1 ± 0.6	4.7 ± 0.6	4.9 ± 0.5
LVEDV (mL)	Sham	0.5 ± 0.1	0.7 ± 0.3	0.7 ± 0.2	0.8 ± 0.3
	10% HG	0.6 ± 0.2	0.7 ± 0.2	0.8 ± 0.4	0.9 ± 0.5
	20% HG	0.7 ± 0.2	0.7 ± 0.3	0.9 ± 0.4	0.8 ± 0.3
	CMs	0.6 ± 0.2	0.6 ± 0.3	0.8 ± 0.4	1.0 ± 0.4
	CMs + 10% HG	0.6 ± 0.2	0.9 ± 0.2	1.2 ± 0.4	1.0 ± 0.4
	CMs + 20% HG	0.6 ± 0.2	0.8 ± 0.2	0.8 ± 0.2	1.0 ± 0.3
Akinetic length (%)	Sham	0	24.6 ± 5.5	24.3 ± 3.9	24.3 ± 3.8
	10% HG	0	26.7 ± 2.6	25.3 ± 2.4	25.0 ± 4.2
	20% HG	0	21.1 ± 3.0	22.8 ± 4.4	22.7 ± 2.9
	CMs	0	22.7 ± 4.5	21.5 ± 3.0	23.5 ± 5.0
	CMs + 10% HG	0	24.6 ± 3.0	24.7 ± 4.3	22.6 ± 3.8
	CMs + 20% HG	0	23.1 ± 5.2	21.3 ± 3.7	21.5 ± 1.9

**Table 3:** Cardiac magnetic resonance imaging data after treatment.

Parameters (mean ± SD)	Group	4 W
LVEF (%)	Sham	33.8 ± 4.7
	10% HG	32.7 ± 12.0
	20% HG	37.6 ± 10.0
	CMs	45.5 ± 3.0
	CMs + 10% HG	41.8 ± 2.8
	CMs + 20% HG	50.5 ± 4.1
LVEDV (µL)	Sham	251.4 ± 69.1
	10% HG	301.0 ± 40.7
	20% HG	294.5 ± 44.3
	CMs	437.0 ± 138.1
	CMs + 10% HG	351.4 ± 152.9
	CMs + 20% HG	357.8 ± 103.5
LVESV (µL)	Sham	168.0 ± 51.0
	10% HG	207.8 ± 61.3
	20% HG	202.2 ± 73.4
	CMs	237.4 ± 75.7
	CMs + 10% HG	203.0 ± 83.0
	CMs + 20% HG	176.0 ± 49.9

## Discussion

In this research, we demonstrated that HG concentration influenced local retention of injected hiPSC-CMs in cardiac tissue. Using a rat MI model for isolated cell transplantation of hiPSC-CMs suspended in 20% HG, we observed the highest tissue retention efficiency of transplanted CMs and a significant improvement in cardiac function. This suggests that the high viscosity of 20% HG likely suppressed diffusion and washout of injected cells, thereby enhancing their retention at the injected region and leading to improved functional outcomes.

In our previous study^20^, we demonstrated that leakage from needle injection points in chicken meat increased with both excessively high and low viscosity of injected HG solutions, highlighting an optimal intermediate concentration that minimizes leakage. Similarly, in this study using static rat myocardial tissue, the 10% HG group showed the least leakage from the insertion point, consistent with the prior findings. Additionally, we found that in fresh static myocardial tissue, diffusion within the tissue in intravascular and interstitial manners more significantly influenced local fluid retention than direct leakage from the insertion point, which is a new observation in this study.

Considering tissue diffusion, the presence or absence of intramyocardial tissue pressure changes during myocardial contraction would be crucial. Significant differences in diffusion dynamics of solutions may occur between beating and non-beating myocardium. In vivo ink injection tests using beating myocardium revealed that the 20% HG solution, with its higher viscosity, tended to remain more efficiently at the injection site compared to lower concentrations (0% and 10% HG). From this observation, it can be inferred that in beating myocardial tissue with significant intramyocardial pressure changes, higher viscosity and concentration of HG solutions are likely to enhance retention around the injection site. On the other hand, the injection of a high-viscosity and large-volume HG solution may potentially impose some form of stress on the heart. Further careful evaluation is necessary to determine the optimal viscosity and injection volume.

For more efficient cardiac regenerative medicine, it is essential not only to consider leakage from insertion points of therapeutic injection fluids but also to evaluate tissue diffusion dynamics, especially regarding the impact of internal pressure in target tissues. This suggests that optimal concentrations of HG solutions as therapeutic injection additives may vary depending on tissue conditions (e.g. live tissue, necrotic tissue, fibrotic tissue and so on), and tissue types (e.g. heart, liver, skeletal muscle). In regenerative medicine, it is crucial to adjust the viscosity of the injection solution to ensure it remains in the host tissue environment as long as possible. For this purpose, HG, which allows for easy adjustment of viscosity by modifying the solution concentration, is considered a highly useful biomaterial.

In this transplantation study, myocardial cell retention within the transplanted tissue was highest in the 20% HG group, which likely contributed to the recovery of cardiac function. However, it remains unclear whether the therapeutic mechanism was due to the direct contraction of the transplanted myocardial cells or the paracrine effects. Previous reports have indicated that myocardial cells can serve as a paracrine source in cardiac regenerative therapies^21–23^. In the context of paracrine effects improving cardiac function, it is believed that a higher number of retained transplanted cells, which act as a paracrine source, would enhance the therapeutic effect. Therefore, conditions that temporarily increase the retention of transplanted cells could amplify the therapeutic benefits. This finding may be valuable for optimizing the therapeutic efficacy of cardiac regenerative therapies. We also observed the highest engraftment of CMs near the injection site in the 20% HG group (Fig. 3B). However, in the Indian ink injection experiment, no significant difference in the degree of tissue diffusion was observed between the 10% HG and 20% HG groups (Fig. 2C). This suggests that, as reported in a previous study^24^, differences in the extracellular environment, such as the extracellular matrix surrounding the injected CMs, may have influenced the proliferation of CM after injection. This aspect requires further investigation in future studies.

The use of cardiac spheroids has been reported as a method to enhance the retention of transplanted CMs within the tissue^25^. Here, it is reported that the size of cardiac spheroid affects survival rates of transplanted cells and tissue diffusion dynamics when administered into the coronary artery^26,27^. With this in mind, and to avoid bias in addressing the primary objective of this study—namely, how the biomaterial suspension itself affects the survival and dynamics of transplanted cells—we opted to perform the injection using dissociated single cells rather than spheroids. This approach allowed us to demonstrate, as hypothesized, that the biomaterial itself can influence the in-tissue dynamics and therapeutic efficacy. On the other hand, the somewhat less pronounced therapeutic effect observed in this study compared to similar previous reports may be related to our decision not to use cell configurations such as spheroids or cell sheets. Moving forward, it will be necessary to investigate whether HG concentration affects cell retention and therapeutic efficacy not only for single cells but also for other transplanted cell forms that have been previously reported.

In addition to direct surgical transplantation via epicardium, catheter-based needle endomyocardial injection offer an alternative approach for cell transplantation^28^. Given its ease of concentration and viscosity adjustment, HG is anticipated to cause fewer issues with injection route limitation or cell transplantation-related complications compared to conventional biomaterials. This makes HG a potentially suitable solvent for catheter-based cell transplantation therapies.

This study has limitations as described below. First, controlling leakage from the injection site in beating myocardium through HG concentration adjustments is crucial for future applications, such as preventing embolic complications during catheter-based cell transplantation from the endocardium. However, while we were able to quantify leakage from the injection site in ex vivo myocardial tissue, we could not measure leakage in the in vivo beating heart within the thoracic cavity. This was because we could not devise a method to distinguish and measure the exact amount of leakage at the injection site from the surrounding blood. Future work should focus on developing precise quantification methods, potentially using radioisotopes (RI), to accurately measure leakage from the injection site in beating myocardium. Second, due to concerns about potential damage to transplanted cells caused by excessive resistance during solution injection under the experimental conditions of this study, we were unable to investigate the efficiency of CM retention and therapeutic efficacy at HG concentrations higher than 20% in vivo. However, we were able to demonstrate that differences in viscosity corresponding to varying HG concentrations influence CM retention and therapeutic outcomes. Nevertheless, we cannot conclude whether 20% is the optimal concentration for maximum effect, and this will require further investigation. Finally, due to the limitations of our research framework, we had to conclude the cardiac function evaluation one month after cell transplantation. We speculate that if a longer observation period had been conducted, the therapeutic superiority of combining 20% HG might have been demonstrated more clearly. For future preclinical studies using large animals, we should plan to set a longer observation period for clinical implementation of this strategy.

## Methods

### Preparation of HG solution

#### Production of HG

The HG sample is a polypeptide created by hydrolyzing porcine skin gelatin produced through acid or alkali extraction methods. Specifically, the sample is beMatrix™ gelatin from Nitta Gelatin Inc. (Yao, Japan), and its detailed properties have been reported in previous studies^20,29^. The beMatrix™ gelatin was dissolved in phosphate-buffered saline (PBS) to prepare 0%, 10% and 20% (w/v) HG solutions, with the pH adjusted to 7.4 ± 0.1 using NaOH.

#### Evaluation of HG and Indian ink viscosity

Indian ink without colloid was purchased from Thermo Fisher Scientific (Waltham, MA USA; Cat. No. J61007.AP). To prepare the Indian ink solutions with various HG concentrations, we first prepared a 10% Indian ink solution by diluting the ink in PBS. Then, we mixed this 10% Indian ink solution with HG solutions at double the desired final HG concentration in a 1:1 ratio. The viscosity of the 10% HG and 20% HG solutions was measured using a rheometer (MCR302, Anton Paar, Graz, Austria) with a cone-plate geometry (25 mm diameter, 1° angle) at a shear rate of 200 1/s and a temperature of 25 °C. The viscosity value recorded after 1 min of measurement was used. The viscosity of the ink was measured using an Ostwald viscometer (Sibata Scientific Technology Ltd., Soka, Japan; item code: 026300-1). The viscometer was immersed in a warm bath at 30 °C for over 5 min, and 6 ml of the reagent was used for the measurement. The relative viscosity was then calculated by comparing it with the known viscosity of the 10% HG solution.

### Preparation of hiPSC-CMs

#### Maintenance of hiPSCs

We used the hiPSC line (201B6) derived from skin fibroblasts, established at and provided from the Center for iPS Cell Research and Application, Kyoto University^30^. The hiPSCs were cultured as previously described with modifications^31^. In brief, the hiPSCs were cultured and maintained in StemFit AK02N medium (Ajinomoto, Tokyo, Japan). Upon reaching confluence, the cells were dissociated using TrypLE Select (Thermo Fisher Scientific) and dissolved in PBS containing 0.5 mM ethylenediaminetetraacetic acid (EDTA) (1:1). They were then subcultured every 7 days at a density of 5000–8000 cells/cm^2^ in AK02N medium with iMatrix-511 silk (FUJIFILM Wako Pure Chemical Corporation, Osaka, Japan) (0.125 µg/cm^2^, uncoated laminin fragment)^32^ and ROCK inhibitor (Y-27632, 10 µM, FUJIFILM Wako).

#### Differentiation of hiPSCs into CMs

For cardiovascular cell differentiation, dissociated hiPSCs were seeded onto Matrigel-coated plates (1:60 dilution) at a density of 360,000–410,000 cells/cm^2^ in AK02N and Y-27,632 (10 µM). After 1–2 days, the cells reached confluence, and the hiPSCs were covered with Matrigel (1:60 dilution in AK02N) the day before induction. The AK02N medium was replaced with RPMI + B27 medium (RPMI 1640, Thermo Fisher; 2 mM L-glutamine, Thermo Fisher; B27 supplement without insulin, Thermo Fisher) mixed with 100 ng/mL Activin A (R&D, Minneapolis, MN, USA) and 5 µM CHIR99021 (Tocris Bioscience, Bristol, UK). This day was defined as differentiation day 0 (d0). After 24 h, the medium was changed to RPMI + B27 medium containing 10 ng/mL bone morphogenetic protein 4 (BMP4; R&D) and 10 ng/mL basic fibroblast growth factor (bFGF, FUJIFILM Wako) (d1). No medium change was performed for the next 4 days. For CM induction, the medium was replaced on d5 with RPMI + B27 medium containing 2.5 µM IWP4 (Stemgent, Cambridge, MA, USA) and 5 µM XAV939 (Merck, Kenilworth, NJ, USA). From then until d13, the medium was replaced every other day with RPMI 1640 insulin plus medium. Beating CMs appeared between d11 and d13.

The hiPSC-CMs were prepared at RIKEN, Kobe, Japan and transferred to Kyoto University, Kyoto, Japan using iP-TEC® live cell transportation system (SANPLATEC CO., Ltd., Osaka, Japan) within 2 h. On day 13 of differentiation, the cardiovascular cells were dissociated using Accutase™ (Nacalai Tesque, Inc, Kyoto, Japan) and subjected to in vivo transplantation experiments and flow cytometry analysis.

#### Flow cytometry

On d13, hiPSC-derived CMs were dissociated and stained with the LIVE/DEAD fixable Aqua dead cell staining kit (Thermo Fisher) to identify dead cells. Anti-VE-cadherin (APC conjugated, clone 55-7h1, 1:100) (BD, Franklin Lakes, NJ, USA) was used as a cell surface marker. The staining solution contained FC buffer [5 mM-EDTA + 5% fetal bovine serum (FBS)/PBS]. The cells were then fixed with PBS containing 4% paraformaldehyde (PFA). Intracellular protein staining was performed using APC-labeled anti-cardiac troponin T (cTnT) (clone 13-11) (Thermo Fisher) at a 1:50 concentration using Zenon technology (Thermo Fisher). The staining solution contained PBS with 5 mM EDTA, 5% FBS, and 0.75% saponin (Sigma-Aldrich, St. Louis, MO, USA). Stained cells were analyzed using CytoFLEX S (Beckman Coulter, Brea, CA, USA). Data were collected from at least 10,000 events and analyzed using CytExpert software (Beckman Coulter). The ratio of hiPSC-CMs marked by cTnT among total cells was 76.8 ± 10.4% in total cells (*n* = 7).

### Injection experiments

#### Animals

All animal experiment protocols were approved by the Animal Experimentation Committee of Kyoto University (#MedKyo 23155). All animal experiments were conducted in accordance with Japanese law and the “*Guide for the Care and Use of Laboratory Animals*” of the US National Research Council. For ex vivo experiments and in vivo Indian ink injection experiments, male Jcl: SD rats purchased from CLEA Japan (Tokyo, Japan) were used. For in vivo hiPSC-CM injection experiments, male athymic nude rats (F344/NJcl-rnu/rnu, 12–16 weeks old) purchased from CLEA Japan were used. All rats were housed under controlled conditions. For euthanasia, all rats were rapidly administered 40 ml of potassium chloride solution intravenously under 5% isoflurane (Pfizer, Tokyo, Japan) inhalation. This study the study is reported in accordance with ARRIVE guidelines (https://arriveguidelines.org).

#### Ex vivo experiments

The animals were intubated with a 16-gauge angiocath (TERUMO, Tokyo, Japan) and mechanically ventilated under general anesthesia with 2% isoflurane. After euthanasia, hearts were excised and HG solutions of varying concentrations and injection volumes were injected into the fresh rat left ventricular myocardium after the spontaneous beating stopped. A 30-G needle was used for the injections, with HG solution concentrations of 0% HG, 10% HG, and 20% HG, and injection volumes of 100 µL, 200 µL, and 300 µL, respectively. The hearts were divided into two parts longitudinally, and 27 heart samples were used in total. The leakage from the cross-section refers to the amount of solution leaking from the myocardial cross-section when the heart is bisected along its longitudinal axis. The amount of leakage was quantified by absorbing the leaked solution from the injection site and the tissue cross-section of the left ventricle with filter paper, and the weight change of the filter paper was measured.

#### In vivo Indian ink injection experiments

Three different concentrations of HG + Indian ink solutions were prepared: 0% HG + Indian ink (*n* = 4), 10% HG + Indian ink (*n* = 5), and 20% HG + Indian ink (*n* = 5), then used for in vivo injection. The animals were intubated and mechanically ventilated under general anesthesia with 2% isoflurane as described in "Ex vivo experiments". Hearts were exposed through left thoracotomy and pericardiotomy. Indian ink solution was injected using a 30-G blunt needle (GL Sciences replacement needle for Hamilton syringe, Tokyo, Japan; model N730). A volume of 300 µL was injected into the myocardium at the middle part of the anterior wall of left ventricle, and the hearts were excised immediately after injection. The hearts were fixed in 4% PFA for 24 h.

#### In vivo hiPSC-CM injection experiments

##### Induction of myocardial infarction (MI)

The animals were anesthetized as descried in "In vivo Indian ink injection experiments". MI was induced by permanent ligation of the left anterior descending coronary artery. The induction of MI was confirmed by the pallor of the myocardial surface^33^. In hiPSC-CM transplantation study, a total of 85 rats underwent MI induction. Twenty rats (24%) experienced early death from MI. Rats with a left ventricular (LV) fractional shortening (FS) of 30% or higher on echocardiogram performed 7 days after ligation were excluded from subsequent experiments. Six rats (7%) were excluded because of insufficient MI; thus, 59 rats were enrolled in the experiments. All experiments were conducted with double-blinded manner.

##### Assessment of Engraftment following hiPSC-CM transplantation

In this experiment, 18 eligible MI-induced rats were enrolled and randomly assigned. Three rats (17%) died immediately after the CM transplantation, and 15 rats were finally evaluated: a group transplanted with cardiomyocytes dissolved in regular Ringer’s solution (CMs; *n* = 4), a group transplanted with cardiomyocytes dissolved in 10% HG solution (CMs + 10% HG; *n* = 6), and a group transplanted with cardiomyocytes dissolved in 20% HG solution (CMs + 20% HG; *n* = 5). One week after myocardial infarction induction, hiPSC-CMs were dissociated into single cells, and 1.0 × 10^7^ cells were prepared in solutions dissolved in regular Ringer’s solution (CMs), 10% HG solution (CMs + 10% HG), and 20% HG solution (CMs + 20% HG). To detect engrafted cells post-transplantation, cell nuclei were labeled with Hoechst 33342 (Thermo Fisher; 5 µg/ml, 10 min in the dark at 37 °C) prior to transplantation. Each solution was injected into a single site of the myocardial infarction border zone using a 30-G Hamilton needle (blunt needle) with a volume of 300 µL. The volume of the injection solution for the rat hearts was set following the amount used in previous similar cell transplantation experiments in rats^24^. Hearts were harvested one-week post-transplantation for histological evaluation. Animals were euthanized at the end of the one-week observation period post-transplantation, and graft survival was histopathologically assessed and compared between groups.

##### Functional assessment following hiPSC-CM transplantation

In this experiment, 41 eligible MI-induced rats were enrolled and randomly assigned. Five rats (12%) died immediately after the CM transplantation, and 36 rats were finally evaluated: a sham group that underwent left thoracotomy without transplantation (sham; *n* = 7), a group transplanted with only 10% HG solution (10% HG; *n* = 5), a group transplanted with only 20% HG solution (20% HG; *N* = 6), as well as the three previously mentioned groups: CMs (*n* = 7), CMs + 10% HG (*n* = 5), and CMs + 20% HG (*n* = 6). The transplantation procedure was performed in the same manner as described in "Assessment of engraftment following hiPSC-CM transplantation". For post-transplant cardiac function evaluation, echocardiogram was performed before MI induction (preMI), before cell transplantation (preTx), and at 2 and 4 weeks post-transplantation, respectively. Cardiac magnetic resonance imaging (MRI) was conducted at 4 weeks post-transplantation.

### Cardiac functional assessment

#### Echocardiogram

To evaluate overall cardiac function and left ventricular (LV) size, echocardiogram was conducted using the Vivid 7 system (GE Healthcare, Waukesha, WI, USA) equipped with an 11-MHz imaging transducer (GE 10 S ultrasound probe, GE Healthcare). Echocardiogram was performed in a double-blinded manner. End-diastolic dimension of LV (LVDd), End-systolic dimension of LV (LVDs), and End-diastolic volume of LV (LVEDV) were measured using M-mode examination. LV end-diastolic circumference (CIRCd) and the length of akinetic region (LAR) were recorded and measured using B-mode examination. The values were calculated as follows:

$$\begin{gathered} {\text{Fractional}}\;{\text{shortening}}\;({\text{FS}})\;{\text{(\% ) }}=\left( {{\text{LVDd }} - {\text{ LVDs}}} \right)/{\text{LVDd }} \times {\text{ }}100 \\ {\text{Akinetic}}\;{\text{lesion}}\;({\text{AL}})\;(\% )={\text{LAR / CIRCd }} \times 100  \\ \end{gathered}$$

#### Cardiac MRI

At the end of the 4-week observation period for each group, under general anesthesia with 2% isoflurane (Pfizer), MRI (7-T BioSpec 70/20 USR; Bruker Biospin, Ettlingen, Germany) was used for cardiac functional evaluation. LVEDV (left ventricular end-diastolic volume) and LVESV (left ventricular end-systolic volume) were obtained using ImageJ software, and LVEF (left ventricular ejection fraction) was calculated using the following formula:

$${\text{LVEF}}\;(\% )=100{\text{ }} \times {\text{ }}({\text{LVEDV }} - {\text{ LVESV}}){\text{ /(LVEDV)}}.$$

### Histological analysis

Heart samples fixed with 4% PFA were prepared as cross-sections. For in vivo Indian ink injection, the cross-section at the injection site was defined as the middle plane, halfway between the middle plane and the apex was defined as the apex plane, and the cross-section equidistant from the middle plane to the base was defined as the base plane. Photographs of each cross-section were taken, and the ink-stained area was measured using ImageJ (National Institutes of Health, Bethesda, MD, USA). To evaluate the diffusion pattern of the injected solution within the cardiac tissue, paraffin-embedded heart tissue sections were cut at a thickness of 4 micrometers and stained with hematoxylin and eosin.

For in vivo hiPSC-CM injection, fixed hearts were paraffin-embedded and processed into 4-µm thick paraffin-embedded sections using standard methods. Immunostaining for cTnT was performed using a polyclonal rabbit anti-cardiac troponin-T antibody (ab45932; Abcam, Cambridge, UK). Visualization was carried out using the conventional HRP/DAB method with Avidin-biotin-peroxidase complex. We defined the engraftment area as the region where cTnT-positive cells were observed within the range of Hoechst 33342-positive cells (Supplementary Fig. 1). All immunostained sections were analyzed using an all-in-one microscopy (BZ-X810, Keyence, Osaka, Japan). To more accurately quantify the CM-positive areas, we prepared gated images using a ‘Color Deconvolution 1.7’ plugin of Fiji ImageJ. This plugin provides a method for quantifying color information in images by separating specific color components^34,35^. Then we quantified the percentage of CM-positive areas using Fiji ImageJ. Specifically, the red, green, and blue (RGB) original image obtained by a BZ-X810 microscope (brightfield) was used. When Color Deconvolution 1.7 was applied to this image, the absorbance values (ranging from 0 to 1) for the RGB components were determined using a set of experimentally decided ‘built-in’ staining vectors. Images with absorbance values of Red: 0.268, Green: 0.570, Blue: 0.776 were chosen. Subsequently, the same Brightness/Contrast and Threshold settings were applied to all images using functions of ImageJ. The values of the Brightness/Contrast and Threshold settings were determined visually to identify optimal values. For each animal with confirmed engraftment, we quantified the cTnT-positive area fraction in two random fields for each of two slides, spaced 20 μm apart near the injection site. The area ratio (%) of regions stained with cTnT under a single field of view was then measured.

### Statistical analysis

Data with non-normal distribution were presented as medians (interquartile range [IQR]), while data with normal distribution were presented as means ± standard deviation (SD). All data analyses were performed using GraphPad Prism 8.3.0 (GraphPad Software, Inc., Boston, MA, USA). Kruskal–Wallis test with post-hoc group comparisons conducted using Dunn’s multiple comparison test, or one-way analysis of variance (ANOVA) with Tukey’s test as post-hoc was correctly applied for comparing more than two groups. For time-course comparisons between groups, two-way ANOVA with Geisser–Greenhouse correction was used with post-hoc group comparisons using Tukey’s multiple comparison test. In cases of missing values, a mixed-effects model with restricted maximum likelihood (REML) was employed. A P-value of less than 0.05 was considered statistically significant.

## Electronic supplementary material

Below is the link to the electronic supplementary material.

Supplementary Material 1

## Data Availability

Data is provided within the manuscript.
