# Supplementary material for: Retention of locally injected human iPS cell-derived cardiomyocytes into the myocardium using hydrolyzed gelatin
Source: Sci Rep. 2025 Feb 7;15:4635. doi: 10.1038/s41598-025-87885-w (PMC11806045; doi:10.1038/s41598-025-87885-w)
Supplement: Supplementary file 1 — Supplementary Material 1 [file 41598_2025_87885_MOESM1_ESM.docx]

**Supporting Information**

**Retention of locally injected human iPS cell-derived cardiomyocytes into the myocardium using hydrolyzed gelatin**

Jun Iida^1,2^, Kazuki Kotani^3^, Kozue Murata^1^, Wusiman Maihemuti^1^, Yoshinobu Mandai^3^, Yosuke Hiraoka^3^, Kenji Minatoya^2^, Hidetoshi Masumoto^1,2^

1: Clinical Translational Research Program, RIKEN Center for Biosystems Dynamics Research, Kobe, Japan

2: Department of Cardiovascular Surgery, Graduate School of Medicine, Kyoto University, Kyoto, Japan

3: Biomedical Department, R&D Center, Nitta Gelatin Inc., Yao, Japan


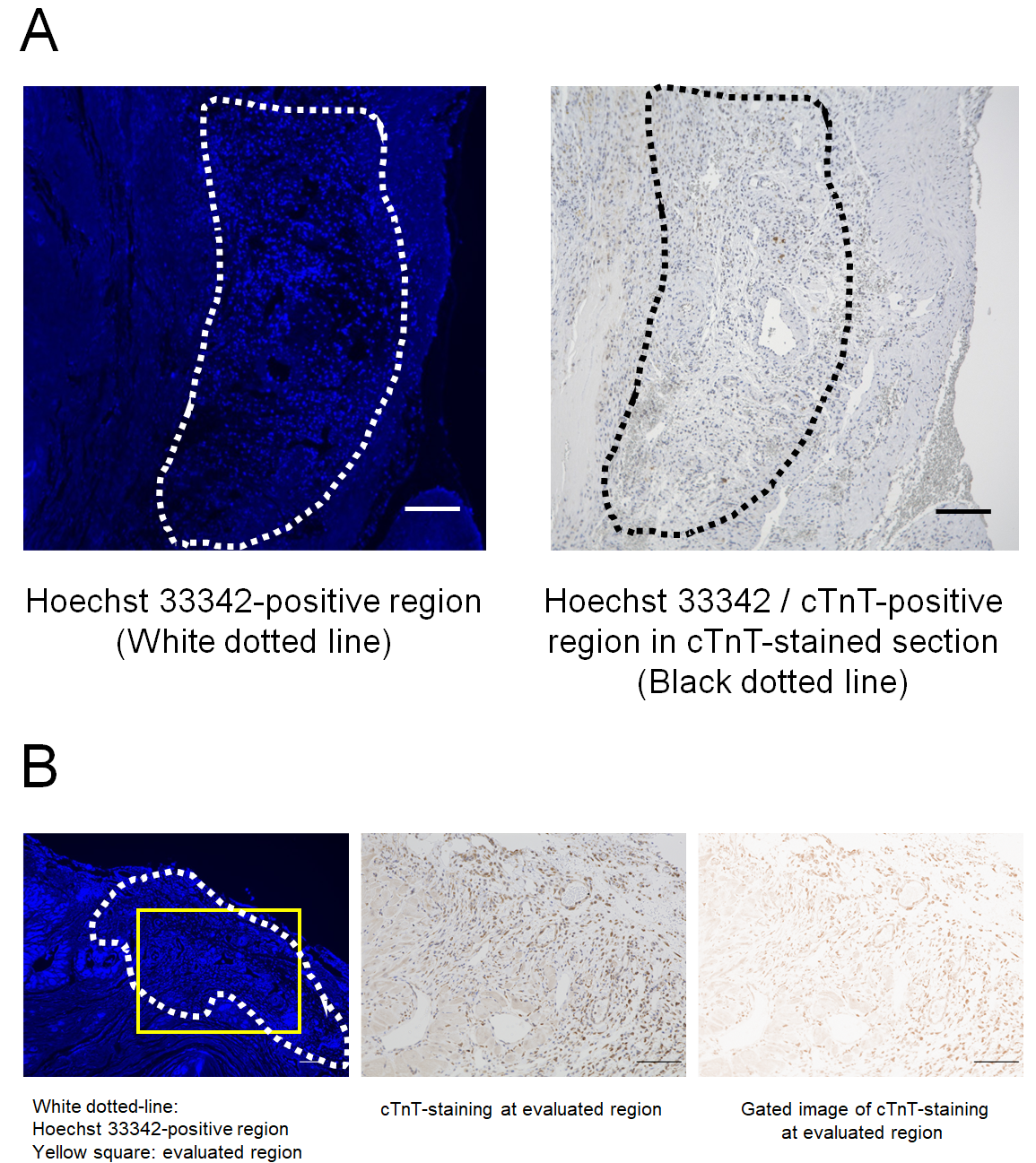


**Supplementary Figure 1: Confirmation of engrafted CMs.**

(**A**) Comparative images of Hoechst 33342-positive region and cTnT-stained section. Scale bars = 100 µm. (**B**) Representative gating processed images. Scale bars = 100 µm.
